# Machine Learning Identifies Variation in Timing of Palliative Care Consultations Among Traumatic Brain Injury Patients

**DOI:** 10.21203/rs.3.rs-4290808/v1

**Published:** 2024-05-02

**Authors:** Carlos A. Aude, Vikas N. Vattipally, Oishika Das, Kathleen R. Ran, Ganiat A. Giwa, Jordina Rincon-Torroella, Risheng Xu, James P. Byrne, Susanne Muehlschlegel, Jose I. Suarez, Debraj Mukherjee, Judy Huang, Tej D. Azad, Chetan Bettegowda

**Affiliations:** The Johns Hopkins University School of Medicine; The Johns Hopkins University School of Medicine; The Johns Hopkins University School of Medicine; The Johns Hopkins University School of Medicine; The Johns Hopkins University School of Medicine; The Johns Hopkins University School of Medicine; The Johns Hopkins University School of Medicine; The Johns Hopkins University School of Medicine; The Johns Hopkins University School of Medicine; The Johns Hopkins University School of Medicine; The Johns Hopkins University School of Medicine; The Johns Hopkins University School of Medicine; The Johns Hopkins University School of Medicine; The Johns Hopkins University School of Medicine

## Abstract

Background and Objective Timely palliative care involvement offers demonstrable benefits for traumatic brain injury (TBI) patients; however, palliative care consultations (PCCs) are used inconsistently during TBI management. This study aimed to employ advanced machine learning techniques to elucidate the primary drivers of PCC timing variability for TBI patients. Methods Data on admission, hospital course, and outcomes were collected for a cohort of 232 TBI patients who received both PCCs and neurosurgical consultations during the same hospitalization. Principal Component Analysis (PCA) and K-means clustering were used to identify patient phenotypes, which were then compared using Kaplan-Meier analysis. An extreme gradient boosting model (XGBoost) was employed to determine drivers of PCC timing, with model interpretation performed using SHapley Additive exPlanations (SHAP). Results Cluster A (n = 86) consisted mainly of older (median [IQR] = 87 [78, 94] years), White females with mild TBIs and demonstrated the shortest time-to-PCC (2.5 [1.0, 7.0] days). Cluster B (n = 108) also sustained mild TBIs but comprised moderately younger (81 [75, 86] years) married White males with later PCC (5.0 [3.0, 10.8] days). Cluster C (n = 38) represented much younger (46.5 [29.5, 59.8] years), more severely injured, non-White patients with the latest PCC initiation (9.0 [4.2, 17.0] days). The clusters did not differ by discharge disposition (p = 0.4) or frequency inpatient mortality (p > 0.9); however, Kaplan-Meier analysis revealed a significant difference in the time from admission to PCC (p < 0.001), despite no differences in time from admission to mortality (p = 0.18). SHAP analysis of the XGBoost model identified age, sex, and race as the most influential drivers of PCC timing. Conclusions This study highlights crucial disparities in PCC timing for TBI patients and underscores the need for targeted strategies to ensure timely and equitable palliative care integration for this vulnerable population.

## INTRODUCTION

Traumatic brain injury (TBI) is the leading cause of disability and mortality from traumatic injuries globally.^1,2^ In the United States alone, over 200,000 people are hospitalized, and nearly 70,000 die from TBI annually.^3^ While neurosurgical interventions can minimize secondary brain injury, the heterogeneous nature of TBI requires complex clinical decision-making, and prognostic challenges further complicate the timely transition to end-of-life care.^4–8^

Palliative care provides active, whole-person care to individuals nearing the end of life to improve the quality of life for patients, families, and caregivers.^9^ As such, palliative care can play a vital role in clarifying and codifying care goals among patients with TBI.^10^ Timely palliative care consultations (PCCs) have been shown to facilitate decision-making about care goals, reduce hospital stay duration and costs, and minimize non-beneficial interventions without increasing mortality rates in patients with TBI.^11–16^ Despite these benefits, integration of palliative care for patients with TBI is limited and varies by sociodemographic and clinical factors, highlighting care discrepancies.^6,17–22^ Optimal integration and timing of palliative care in TBI is a crucial yet severely understudied area that needs of further investigation.^23^

In response to these challenges, this study aims to uncover the primary factors influencing the timing of initial PCCs in patients with TBI. Building on recent efforts to identify TBI phenotypes, we extend these methods to patients with TBI receiving palliative care consultations, and additionally employ advanced machine learning techniques to elucidate the precise impact of each variable.^24–27^ We hypothesize that identifying distinct clusters of patients based on admission characteristics will reveal significant disparities in the timing of initial PCCs. Additionally, we theorize that factors beyond injury and clinical details are responsible for producing these clusters and play a crucial role in the timing of PCC. These insights could help clinicians identify patients with TBI at greatest need for timely palliative care and develop targeted strategies to ensure equitable access, thereby reducing unnecessary interventions and improving the quality of life of these patients and their families.

## METHODS

### Study Design and Population

This retrospective study included adult (≥18 years) patients admitted to Johns Hopkins Medicine hospitals between 2016 and 2022 who received both neurosurgery consultation and PCC during the same hospital admission. Our initial cohort comprised 270 patients, from which we excluded non-TBI admissions (n = 34) and penetrating mechanisms of injury (MOI) (n = 4), resulting in a final study population of 232 patients. The Johns Hopkins Medicine Institutional Review Board approved this study (IRB00309385), and the requirement for informed consent was waived because data was collected retrospectively from electronic health records (EHRs). We used the STROBE cohort checklist when writing our report.^28^

### Data Collection and Preprocessing

Data were extracted from Johns Hopkins Medicine EHRs, encompassing a broad range of variables categorized into admission, hospital course, and outcome variables. Admission variables included demographics, clinical and injury characteristics, radiographic findings, attending neurosurgeon, and care decision details such as a care-limiting directive present on admission and the identity of the surrogate decision-maker (SDM). Importantly, we collected the Glasgow Coma Scale motor sub-scores (mGCS) as a validated alternative to total GCS scores for intubated TBI patients.^29^

Hospital-course variables encompassed intensive care unit (ICU) admission and length of stay, interactions between surrogate decision-makers (SDMs) and providers, and neurosurgical interventions. These interventions were quantified using the Therapy Intensity Level Scale (TILS).^30^ The TILS score was determined by summing the scores for head positioning for intracranial pressure (ICP) management (1 point possible), cerebral perfusion pressure (CPP) therapy (2 points), cerebrospinal fluid (CSF) drainage (3 points), mechanical ventilation (4 points), temperature management (5 points), hyperosmolar therapy (6 points), sedation level (8 points), and decompressive surgery (9 points), where higher scores represent a greater degree of intervention intensity. Outcome variables included discharge disposition, inpatient mortality, and time-to-event data for PCC and mortality. Time-to-event metrics were computed as the number of days from hospital admission to the event using EHR-recorded dates.

Given the high dimensionality of the data relative to the sample size, traditional statistical approaches were deemed inappropriate, leading to the adoption of more sophisticated machine learning techniques.^31^ Data preprocessing involved cleaning, standardizing, and transforming categorical variables into one-hot-encoded indicators to facilitate machine learning algorithm compatibility. No data were missing among continuous variables, while missing data among categorical variables were treated as an additional “unknown” category, where applicable.

### Principal Component Analysis and K-means Clustering

The application of Principal Component Analysis (PCA) and K-means clustering aids in the simplification and categorization of complex patient data, allowing for a more focused analysis of critical factors influencing the timing of palliative care. First, PCA was utilized to reduce the dimensionality of the dataset, facilitating the identification of the most important variables within the extensive dataset. Following dimensionality reduction through PCA, K-means clustering was applied to categorize patients into K distinct groups based on the similarity of their data profiles, enabling the identification of patterns and similarities in patient characteristics and treatment variables. The optimal number of clusters (K) was determined using the gap statistic method **(SUPPLEMENTARY FIGURE 1)**.^32^ Clusters were built solely using admission variables. PCA and K-means clustering were performed using R software (version 4.2.1). PCA plots were visualized using the ggplot2 package (version 3.4.4).

### Statistical Comparison of Clusters

K-means-identified clusters (A, B, and C) were then statistically compared. Categorical variables were analyzed using Pearson’s chi-squared or Fisher’s Exact Test, while continuous variables were analyzed using the Kruskal-Wallis rank sum test because of significant skewness in some variables. Post-hoc pairwise comparisons were adjusted for multiple testing with Benjamini-Hochberg and Bonferroni corrections for overall and pairwise tests, respectively. Kaplan-Meier curves, stratified by cluster, were generated for time-to-PCC and time-to-mortality using the survminer package (version 0.4.9), with differences assessed using a log-rank test. Mortality measured by the survival analysis included death recorded in the patient EHRs at any point between initial admission and censoring. Patients were censored at the most recent patient encounter recorded within their EHR. A small random jitter (between 0 and 1 day) was added to the Kaplan-Meier curves to improve visualization, although test statistics were computed using the original data.

### XGBoost Model and SHAP Analysis

An extreme gradient-boosted decision tree model was built in Python (version 3.10.12) using the XGBoost library (version 1.3.2) to model and understand time-to-PCC. The XGBoost model was selected because tree ensemble methods have been shown to consistently outperform competing methods for regression and classification tasks using tabular data.^33^ Superior performance over a simpler alternative, the Least Absolute Shrinkage and Selection Operator (LASSO) regression model, was confirmed for our analysis via bootstrap testing over 100 replications (p < 0.001). XGBoost hyperparameters, including learning rate, tree depth, and sampling ratios, were tuned by randomized search cross-validation (CV) over 100 replications of 5-fold stratified CV, with final training involving early stopping over 100 iterations.

Interpretability of the XGBoost model was enhanced using SHapley Additive exPlanations (SHAP) values. SHAP values explain the output of machine learning models by decomposing the model predictions into the sum of effects of each variable, providing insights into how each variable influences time-to-PCC. This approach has been previously used to understand machine learning models employed in the context of neurocritical care.^34^ The ten variables with the highest mean absolute SHAP values were considered the most influential features in this analysis. The SHAP Python library (version 0.44.0) facilitated the analysis of the most influential features by generating (1) a summary plot of mean absolute SHAP values, (2) scatter plots of SHAP values for each variable, (3) a heatmap of SHAP interaction values, and (4) scatter plots of important interactions.

## RESULTS

### Study Population

In our cohort of 232 TBI patients who received PCC, the median age was 81 years (IQR = [70, 87]) and 54% (125/232) were male. Most were White (59%, 138/232) and of non-Hispanic ethnicity (94%, 218/232), and the predominant mechanism of injury (MOI) was falls (83%, 193/232). At presentation, 81% (187/232) had both pupils reactive and a median mGCS sub-score of 6 (IQR = [5, 6]). During hospitalization, 81% (187/232) of patients were admitted to the ICU with a median ICU length of stay of 6 days (IQR = [3, 13]) and a median TILS score of 2 (IQR = [1, 5]) prior to PCC. Regarding outcomes, 51% (118/232) experienced withdrawal of life-sustaining treatment (WLST), 38% (88/232) experienced inpatient mortality, and an additional 30% (70/232) were discharged to hospice. The median time from admission to PCC was 5 days (IQR = [2, 10]).

### Characterization and Comparison of Patient Clusters

**Figure 1:**
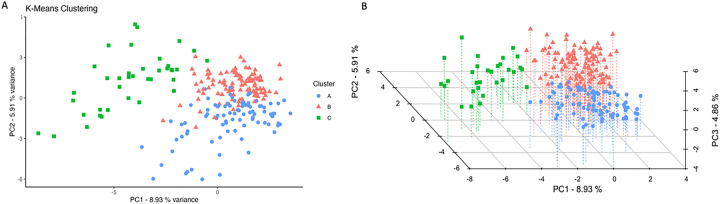
Cluster distribution from K-Means analysis. Panel A depicts a 2-dimensional scatter plot of the first two principal components (PC1 and PC2) that explains 8.9% and 5.9% of the variance, respectively. Each point represents an individual patient, color-coded by the assigned cluster (A, B, C). Panel B extends the analysis into three dimensions with the inclusion of PC3, accounting for an additional 4.9% of the variance and providing a more detailed view of the spatial separation between the clusters. Abbreviations: PC = Principal Component.

Principal component analysis (PCA) captured 8.9%, 5.9%, and 4.9% of the total variance in the first three principal components, respectively. K-means clustering partitioned observations into three distinct clusters, which are visualized using two- and three-dimensional plots (FIGURE 1). Each cluster presented with unique characteristics (TABLE 1). Among the characteristics available at presentation, the clusters differed significantly across 65% (34/52) of all the admission variables recorded **(SUPPLEMENTARY TABLE 1)**.

Among presentation variables, Cluster A was the oldest group (median [IQR] = 87 [78, 94] years) and predominantly consisted of unmarried White females (94% female [83/86], 58% White [51/86], 75% unmarried/widowed [56/86]) with mild TBIs from falls (89%, 78/86) and a high median mGCS sub-score (6 [5, 6]). In comparison, Cluster B was moderately younger (81 [75, 86] years) and comprised mostly married White males (84% male [84/106], 67% white [71/106], 71% married [75/106]), also with mild TBIs from falls (92%, 98/106) and high mGCS sub-score (6 [6, 6]). Finally, Cluster C was much younger (46.5 [29.5, 59.8] years) and included more racial minorities (58% non-White, 22/38) with moderate-severe TBIs, more heterogeneous mechanisms of injury including high-impact MOIs (47%, 22/38), and the lowest median mGCS sub-score (4 [1, 5]). Specifically, Cluster A had the highest percentage of females (p < 0.001); Cluster B had the highest percentage of married patients (p < 0.001) and White patients (p < 0.001); and Cluster C had the most severe injuries with the lowest mGCS sub-scores (p < 0.001) and lowest rate of bilateral reactive pupils at presentation (p < 0.001).

**Figure 2:**
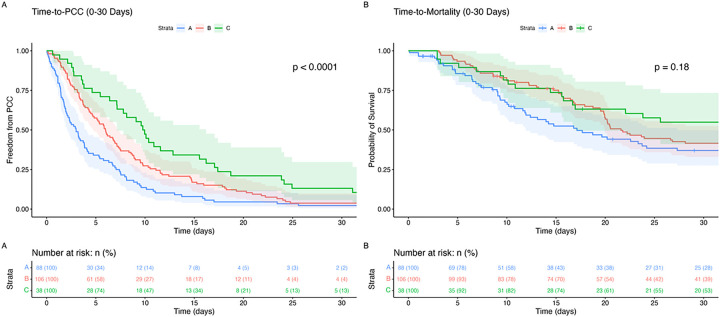
Kaplan-Meier curves for time-to-palliative care consultation (PCC) and time-to-mortality. Panel A shows the time-to-PCC for Clusters A, B, and C, with the shaded areas representing 95% confidence intervals; this curve indicates a statistically significant difference between the clusters (p < 0.0001). Panel B presents the survival analysis for time-to-mortality, with no significant difference observed between the clusters (p = 0.18). The tables below each graph display the number and percent of patients at risk at various time points. The test statistics are computed for all time points post-admission (including post-discharge), but only the first 30 days are visualized. Abbreviations: PCC = Palliative Care Consultation.

Among hospital course and outcome variables, significant differences were noted in ICU admission rates and TILS scores before PCC, with Cluster C having the highest values (97% ICU admission, 37/38, p = 0.021; median TILS score 5.5 [3.0, 13.8], p < 0.001) (TABLE 2). No other recorded hospital course characteristics differed between clusters. Additionally, none of the clusters differed across rates of WLST, inpatient mortality, or discharge disposition (TABLE 3). However, significant differences were observed in time-to-event data. Cluster A had a shorter median time from admission to PCC (2.5 [1.0, 7.0] days) than Cluster B (5.0 [3.0, 10.8] days, p < 0.001) and Cluster C (9.0 [4.2, 17.0] days, p = 0.034), despite no significant difference in time-to-mortality (TABLE 3; FIGURE 2).

### XGBoost Model and SHAP Analysis

**Figure 3:**
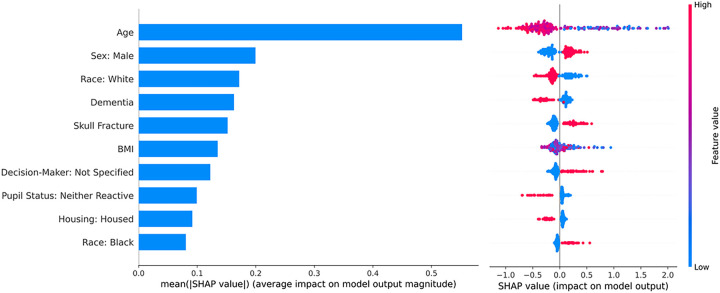
Feature importance for the top ten features identified using SHAP values. The bar chart on the left illustrates the mean absolute SHAP value for each feature, indicating the average impact on the model output magnitude. Age has the highest influence, followed by sex and race. The SHAP value scatter plot on the right shows the impact of each feature value for each individual patient, with color intensity representing the feature value (red for high, blue for low). The clustering of points around high or low SHAP values suggests the directional influence of each feature. Abbreviations: BMI = Body Mass Index, SHAP = SHapley Additive exPlanations.

An XGBoost model, enhanced by SHAP value analysis for model interpretability, identified age as the most important factor influencing time-to-PCC, followed by male sex and White race. Notably, these demographic factors were more influential than clinical factors, such as neurological presentation, radiographic findings, comorbidities, mechanism of injury, and care-limiting directive present on admission (FIGURE 3).

**Figure 4:**
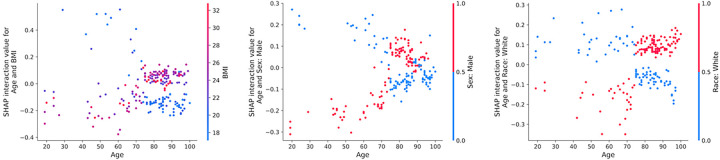
Scatter plots of the three strongest interactions as determined by SHAP interaction values. Points are colored based on the value of the interacting feature, illustrating how the combination of these features impacts the SHAP value. The first plot reveals the interaction between Age and BMI, the second between Age and Male Sex, and the third between Age and White Race. These visualizations highlight the relationships between key features in the model. Abbreviations: BMI = Body Mass Index.

The SHAP analysis offered a visual and comprehensive summary of the impact of each feature on the model **(SUPPLEMENTARY FIGURE 2)**. Further investigation revealed nonlinear relationships between age, race, sex, and BMI, identified through interaction evaluations using a SHAP heatmap **(SUPPLEMENTARY FIGURE 3)** and scatterplots (FIGURE 4). Specifically, the strongest interaction indicated that younger patients with lower BMI were more likely to experience longer time-to-PCC, while the opposite was true for patients with low BMI older than 70 years (FIGURE 4). Other important interactions included Age by Race and Age by Sex, suggesting that younger non-White and younger female patients were also more likely to experience longer time-to-PCC. As with the association between age and BMI, the associations of age with race and sex also reversed directionality for patients older than 70 years (FIGURE 4).

## DISCUSSION

This study significantly advances our understanding of palliative care consultations (PCCs) in patients with traumatic brain injury (TBI) by employing advanced machine learning techniques to identify distinct patient phenotypes and elucidate the primary drivers of PCC timing. Our findings reveal critical variations in the timing of PCC across three clinically meaningful clusters of TBI patients, with demographics, particularly age, playing a more influential role than any other factors, including clinical variables, injury characteristics, or surrogate decision-making details. This study is also the first to link TBI phenotypes to critical decision-making around goals of care (GOC) and the initiation of PCCs, emphasizing how even mild divergences in presentation may hold significance in patient prioritization.

Unsupervised clustering revealed three distinct phenotypic clusters of TBI patients receiving palliative care (FIGURE 1). This approach builds on recent trends in unsupervised stratification of TBI presentations and broadens their scope by incorporating a significantly more comprehensive set of variables.^25–27^ Each cluster identified in our study represents a unique pattern in care delivery, likely reflecting distinct clinical assessments and prognostic evaluations. Cluster A, characterized by early PCC initiation, may imply an early consensus on poor prognosis among the care team. Conversely, Cluster B’s delayed PCC initiation, despite clinical similarities to Cluster A, suggests a potential missed opportunity for earlier palliative involvement. While comparable outcomes between these two clusters argue against the adverse effects of delayed PCC, earlier integration may have eased decision-making and prevented protracted suffering for these patients and families.^11^ Cluster C, with the most severe presentation, highest level of treatment, and latest palliative care involvement, showed outcomes comparable to the other clusters. Survival analysis stratified by each of these clusters aligns with the established literature, suggesting that early integration of palliative care does not adversely affect mortality (FIGURE 2).^13–16^ Together, these phenotypes underscore the differential approaches to palliative care integration, specific to identifiable patient segments.

Given that the identified clusters varied across a wide range of variables, we separately employed a gradient-boosted decision tree model to isolate the individual drivers of differences in PCC timing. Our gradient-boosted decision tree model, enhanced by SHAP value analysis, revealed that demographic factors, especially age, sex, and race, were the predominant drivers of PCC timing variability, outweighing the influence of clinical and injury details (FIGURE 3). The presence of complex interactions between variables in our analysis further supports the appropriateness of our machine learning approach over traditional statistical methods that would have incorrectly assumed independence and linearity of our data (FIGURE 4).

The findings revealed by SHAP value analysis provide potential explanations for the observed variances in PCC timing across the identified patient clusters. For instance, the delayed PCC initiation in Cluster B, which was largely male and moderately younger than Cluster A, suggests a potential ageism bias, where younger, male patients might have been perceived as having a more favorable prognosis or a lesser need for palliative care than the older females in Cluster A. This insight of clinician bias is in line with existing literature that highlights disparities in end-of-life care for patients with TBI.^6,7,21,22,35^ It also supports the increasing recognition that demographics, often unconsciously, influence medical decision-making and care pathways.^36,37^ Our study thus stresses the critical need for increased awareness and the development of strategies to mitigate potential biases in clinical practice.

Palliative care, which is often underutilized for patients with TBI, should be re-envisioned as an essential, proactive component of patient care rather than a reactive or last-resort measure.^17–19^ Its timely integration is crucial not only for its clinical benefits but also for providing compassionate support to patients and families and aiding in the clarification of care goals.^11,12,14,15,23,38^ To this end, increasing the availability of palliative services for patients with TBI and developing clinical triggers tied to influential drivers of PCC timing could offer a systematic method to enhance consultation processes, moving them upstream to play a preventative role. Beyond standardized triggers, enhancing detection of palliative needs warrants policy efforts expanding access and reducing existing disparities.^39^ Current lack of adherence to the palliative care guidelines established by the American College of Surgeons Trauma Quality Improvement Program (TQIP) underscores the need for more effective implementation strategies and highlights the importance of tailoring these guidelines to address the diverse needs of TBI patients.^40,41^ Explicitly integrating social determinants of health frameworks into TBI management guidelines could mitigate biases and barriers to equitable care.^40^ Similarly, specialized training and education addressing implicit biases may increase awareness of disparate palliative integration.^42,43^ Implementing communication enhancements and decision aids could also facilitate more uniform, patient-centered decision-making.^42,44^ Ultimately, realizing timely, compassionate palliative care for all TBI patients requires multifaceted initiatives targeting clinical systems, education, and policy.

### Limitations

The retrospective observational nature of our study limits our ability to draw causal conclusions and introduces the possibility of unmeasured confounding factors. Furthermore, our limited sample size (n = 232) drawn from a single healthcare system may affect the broader applicability of our findings given the variability in TBI presentation and care across different settings. We were unable to confirm the reproducibility of our results with an independent validation cohort; however, we employed rigorous cross-validation and bootstrapping methods during model development to simulate external cohorts. Future studies should aim to directly validate our findings in diverse healthcare environments. Lastly, we were limited to studying patients who received PCCs, but known demographic biases influence the initial provision and acceptance of PCCs and may have further compounded our results.^17,45,46^ Expanding analyses to TBI cohorts not receiving PCCs could offer additional insights into equitable access to palliative services.

## CONCLUSION

This study significantly advances our understanding of palliative care consultations (PCCs) in patients with traumatic brain injury (TBI) and identifies crucial disparities in the timing of PCCs for these patients. Specifically, we demonstrate that injury and clinical characteristics alone inadequately explain PCC timing and revealed that demographic factors, especially age, primarily drive the variability in PCC timing. This finding underscores the need for a more equitable, patient-centered approach that goes beyond relying solely on clinical triggers to ensure timely palliative care. Future efforts should aim to validate these results, develop targeted strategies that incorporate both demographic and clinical factors, address potential biases through clinician education, and promote initiatives that improve access to palliative services for TBI patients.

## Figures and Tables

**Table 1.** Key admission characteristics of patients stratified by patient cluster. This table presents an abridged summary of the variables available at presentation for each cluster. P-values were calculated from the complete data presented in Supplemental Table 1 and show significant differences across clusters, with post-hoc pairwise comparisons adjusted for multiple testing using Bonferroni correction. Abbreviations: GCS = Glasgow Coma Scale, MOI = Mechanism of Injury, MVC = Motor Vehicle Collision, PCC = Palliative Care Consultation, SDM = Surrogate Decision-Maker.

Admission Characteristics	Overall n=232^1^	Patient Cluster			P-value^2^	Post-Hoc Pairwise Cluster Comparisons		
		Cluster A n=86^1^	Cluster B n=106^1^	Cluster C n=38^1^		A vs B^3,4^	A vs C^3,4^	B vs C^3,4^
Key Demographic & Personal Details^5^								
**Age (yrs)**	81.0 (70.0, 87.0)	87.0 (78.0, 94.0)	81.0 (75.0, 86.0)	46.5 (29.5, 59.8)	**<0.001**	***	***	***
**Female Sex**	107 (46%)	83 (94%)	17 (16%)	7 (18%)	**<0.001**	***	***	
**Race**					**<0.001**		**	**
*White*	138 (59%)	51 (58%)	71 (67%)	16 (42%)				
*Black*	49 (21%)	21 (24%)	20 (19%)	8 (21%)				
*Asian*	24 (10%)	11 (12%)	12 (11%)	1 (2.6%)				
*Other/Declined*	21 (9.1%)	5 (5.7%)	3 (2.8%)	13 (34%)				
**Hispanic Ethnicity**	13 (5.6%)	4 (4.5%)	2 (1.9%)	7 (18%)	**0.002**			**
**Body Mass Index**	23.4 (20.3, 26.6)	22.0 (19.0, 25.8)	23.7 (21.0, 26.6)	24.6 (21.7, 28.8)	**0.049**			
**Insurance**					**<0.001**		**	**
*Medicare*	173 (75%)	80 (91%)	89 (84%)	4 (11%)				
*Medicaid*	16 (6.9%)	4 (4.5%)	1 (0.9%)	11 (29%)				
*Commercial*	29 (12%)	4 (4.5%)	10 (9.4%)	15 (39%)				
*Uninsured*	14 (6.0%)	0 (0%)	6 (5.7%)	8 (21%)				
**Key Clinical Details** ^5^								
**GCS Motor**	6.0 (5.0, 6.0)	6.0 (5.0, 6.0)	6.0 (6.0, 6.0)	4.0 (1.0, 5.0)	**<0.001**	*	***	***
**Bilateral Reactive Pupils**^5^	187 (81%)	67 (76%)	96 (91%)	24 (63%)	**<0.001**	*		***
**Most Frequent Mechanisms of Injury** ^5^								
**Fall**	193 (83%)	78 (89%)	98 (92%)	17 (45%)	**<0.001**		***	***
**MVC or Pedestrian vs MVC**	18 (7.8%)	0 (0%)	2 (1.8%)	16 (42%)	**<0.001**		***	***
**Unknown/Unclear MOI**	35 (15%)	17 (19%)	15 (14%)	3 (7.9%)	0.2			
**Most Frequent Radiographic Findings** ^5^								
**Midline Shift (mm)**	0.0 (0.0, 3.2)	0.0 (0.0, 7.0)	0.0 (0.0, 1.8)	0.0 (0.0, 3.0)	**0.003**	**		
**Subdural Hematoma**	157 (68%)	61 (69%)	69 (65%)	27 (71%)	0.7			
**Subarachnoid Hemorrhage**	118 (51%)	46 (52%)	43 (41%)	29 (76%)	**<0.001**		*	***
**Skull Fracture**	67 (29%)	18 (20%)	26 (25%)	23 (61%)	**<0.001**		***	***
**Most Frequent Comorbid Conditions** ^5^								
**Hypertension**	147 (63%)	65 (74%)	77 (73%)	5 (13%)	**<0.001**		***	***
**Dementia**	61 (26%)	32 (36%)	29 (27%)	0 (0%)	**<0.001**		***	***
**Diabetes**	56 (24%)	14 (16%)	40 (38%)	2 (5.3%)	**<0.001**	**		***
**Smoking (Past or Present)**	88 (38%)	23 (26%)	53 (50%)	12 (32%)	**0.002**	**		
**Antithrombotic Medication Use**	112 (48%)	43 (49%)	68 (64%)	1 (2.6%)	**<0.001**		***	***
**Key Decision-Making Details** ^5^								
**Patient Incapacitated**	91 (39%)	32 (36%)	39 (37%)	20 (53%)	0.2			
**Existing Carelimiting Directive**	85 (37%)	42 (48%)	36 (34%)	7 (18%)	**0.005**		**	
**Most Frequent SDMs** ^5^					**<0.001**	**	**	**
*Spouse*	52 (22%)	5 (5.7%)	40 (38%)	7 (18%)				
*Child*	91 (39%)	52 (59%)	36 (34%)	3 (7.9%)				
*Parent*	12 (5.2%)	0 (0%)	0 (0%)	12 (32%)				
*None Specified*	46 (20%)	12 (14%)	23 (22%)	11 (29%)				

1 Median (IQR); n (%)

2 Kruskal-Wallis rank sum test; Pearson's Chi-squared test; Fisher's Exact Test

3 Significance Codes: [0, 0.001]

‘***’ , (0.001, 0.01]

‘**’ , (0.01, 0.05]

‘*’ , (0.05, 1] ‘ ’

4 Bonferroni correction for post-hoc pairwise comparisons

5 Complete details included in Supplementary Table 1

**Table 2.** Hospital course characteristics prior to PCC of patients stratified by patient cluster. This table details interventions, ICU course, and SDM-Provider interactions prior to PCC for each cluster. The p-values are adjusted for multiple comparisons, with significance codes indicating the level of statistical significance for each comparison. Abbreviations: ICU = Intensive Care Unit, PCC = Palliative Care Consultation, SDM = Surrogate Decision-Maker.

Hospital Course Characteristics	Overall n=232^1^	Patient Cluster			Adjusted P-value^2,3^	Post-Hoc Pairwise Cluster Comparisons		
		Cluster A n=86^1^	Cluster B n=106^1^	Cluster C n=38^1^		A vs B^4,5^	A vs C^4,5^	B vs C^4,5^
Interventions Prior to PCC								
**Therapy Intensity Level Score (0–38)**	2.0 (1.0, 5.0)	1.0 (1.0, 4.0)	1.0 (1.0, 3.0)	5.5 (3.0, 13.8)	**<0.001**		***	***
*Medical Therapy Subscore (0–22)*^6^	1.0 (1.0, 3.0)	1.0 (1.0, 2.0)	1.0 (1.0, 2.0)	4.0 (3.0, 6.8)	**<0.001**		***	***
*Invasive Therapy Subscore (0–16)*^7^	0.0 (0.0, 0.0)	0.0 (0.0, 0.0)	0.0 (0.0, 0.0)	0.0 (0.0, 7.0)	**0.018**		*	**
**ICU Course**								
**Admitted to ICU**	187 (81%)	65 (74%)	85 (80%)	37 (97%)	**0.021**		**	*
**Total Length of ICU Stay (Days)**	6.0 (3.0, 13.0)	4.0 (2.0, 10.0)	5.0 (3.0, 13.0)	12.0 (6.0, 22.0)	**0.002**		***	**
**SDM-Provider Interactions Prior to PCC**								
**Num. of SDM-Provider Interactions**	7.0 (4.0, 13.0)	6.0 (4.0, 11.0)	7.0 (3.2, 12.8)	9.5 (6.0, 17.8)	0.10			
**Num. of Physicians Interacting with SDM**	3.0 (2.0, 5.0)	3.0 (2.0, 4.0)	3.0 (2.0, 5.0)	4.0 (2.2, 6.0)	0.5			
**Num. of Relatives Involved in Interactions**	2.0 (1.0, 3.0)	2.0 (1.0, 2.0)	2.0 (1.0, 3.0)	2.0 (1.0, 3.0)	0.2			
**Care-limiting Directive Updated**	172 (74%)	65 (74%)	82 (77%)	25 (66%)	0.5			
**Evidence of Disagreement**	6 (2.6%)	2 (2.3%)	2 (1.9%)	2 (5.3%)	0.6			
**Ethics Committee Involvement**	13 (5.6%)	5 (5.7%)	7 (6.6%)	1 (2.6%)	0.8			

1 Median (IQR); n (%)

2 Kruskal-Wallis rank sum test; Pearson's Chi-squared test; Fisher's Exact Test

3 Benjamini-Hochberg correction for multiple testing

4 Significance Codes: [0, 0.001]

‘***’ , (0.001, 0.01]

‘**’ , (0.01, 0.05]

‘*’ , (0.05, 1] ‘ ’

5 Bonferroni correction for post-hoc pairwise comparisons

6 Medical Therapy Subscore = Head Positioning for ICP (1) + CPP Therapy (2) + Temperature Management (5) + Hyperosmolar Therapy (6) + Sedation Level (8)

7 Invasive Therapy Subscore = CSF Drainage (3) + Mechanical Ventilation (4) + Decompressive Surgery (9)

**Table 3.** Outcomes of patients stratified by patient cluster. This table reports patient outcomes and time- to-event measures for each cluster. The p-values are adjusted for multiple comparisons, with significance codes indicating the level of statistical significance for each comparison. Abbreviations: PCC = Palliative Care Consultation, SNF = Skilled Nursing Facility, WLST = Withdrawal of Life-Sustaining Treatment. Significant P-values bolded.

Outcome Characteristics	Overall n=232^1^	Patient Cluster			Adjusted P-value^2,3^	Post-Hoc Pairwise Cluster Comparisons		
		Cluster A n=86	Cluster B n=106^1^	Cluster C n=38^1^		A vs B^4,5^	A vs C^4,5^	B vs C^4,5^
**Patient Outcome**								
**WLST**	118 (51%)	45 (51%)	54 (51%)	19 (50%)	>0.9			
**Inpatient Death**	88 (38%)	35 (40%)	38 (36%)	15 (39%)	>0.9			
**Death or Hospice**	158 (68%)	66 (75%)	70 (66%)	22 (58%)	0.4			
**Discharge Disposition**					0.5			
*Home*	16 (6.9%)	5 (5.7%)	10 (9.4%)	1 (2.6%)				
*SNF/Subacute Rehab*	47 (20%)	14 (16%)	22 (21%)	11 (29%)				
*Acute Rehab*	11 (4.7%)	3 (3.4%)	4 (3.8%)	4 (11%)				
*Hospice*	70 (30%)	31 (35%)	32 (30%)	7 (18%)				
*Inpatient Death*	88 (38%)	35 (40%)	38 (36%)	15 (39%)				
**Time-to-Event**								
**Admission to PCC (days)**	5.0 (2.0, 10.0)	2.5 (1.0, 7.0)	5.0 (3.0, 10.8)	9.0 (4.2, 17.0)	**<0.001**	***	***	*
**Admission to Inpatient Mortality (days)**^6^	15.0 (7.0, 23.0)	11.0 (5.8, 24.8)	17.0 (9.0, 22.0)	14.5 (8.2, 26.5)	0.7			

1 Median (IQR); n (%)

2 Kruskal-Wallis rank sum test; Pearson's Chi-squared test; Fisher's Exact Test

3 Benjamini-Hochberg correction for multiple testing

4 Significance Codes: [0, 0.001]

‘***’ , (0.001, 0.01]

‘**’ , (0.01, 0.05]

‘*’ , (0.05, 1] ‘ ’

5 Bonferroni correction for post-hoc pairwise comparisons

6 Only patients who experienced inpatient mortality were compared here; mortality at any time postadmission was compared by the survival analysis (Figure 4)
